# Association of clinicopathologic and molecular factors with the occurrence of positive margins in breast cancer

**DOI:** 10.1007/s10549-023-07157-x

**Published:** 2023-12-01

**Authors:** Anupama Praveen Kumar, Diego Vicente, Jianfang Liu, Praveen-Kumar Raj-Kumar, Brenda Deyarmin, Xiaoying Lin, Craig D. Shriver, Hai Hu

**Affiliations:** 1 Chan Soon-Shiong Institute of Molecular Medicine at Windber (CSSIMMW), Windber, PA USA; 2 Naval Medical Center, San Diego, CA USA; 3 Murtha Cancer Center Research Program, Department of Surgery, Uniformed Services University of the Health Sciences, Bethesda, MD USA; 4 Department of Surgery, Walter Reed National Military Medical Center, Bethesda, MD USA

**Keywords:** Margin status, Breast cancer, TCGA, RNA-Seq

## Abstract

**Purpose:**

To explore the association of clinicopathologic and molecular factors with the occurrence of positive margins after first surgery in breast cancer.

**Methods:**

The clinical and RNA-Seq data for 951 (75 positive and 876 negative margins) primary breast cancer patients from The Cancer Genome Atlas (TCGA) were used. The role of each clinicopathologic factor for margin prediction and also their impact on survival were evaluated using logistic regression, Fisher’s exact test, and Cox proportional hazards regression models. In addition, differential expression analysis on a matched dataset (71 positive and 71 negative margins) was performed using Deseq2 and LASSO regression.

**Results:**

Association studies showed that higher stage, larger tumor size (T), positive lymph nodes (N), and presence of distant metastasis (M) significantly contributed (*p* ≤ 0.05) to positive surgical margins. In case of surgery, lumpectomy was significantly associated with positive margin compared to mastectomy. Moreover, PAM50 Luminal A subtype had higher chance of positive margin resection compared to Basal-like subtype. Survival models demonstrated that positive margin status along with higher stage, higher TNM, and negative hormone receptor status was significant for disease progression. We also found that margin status might be a surrogate of tumor stage. In addition, 29 genes that could be potential positive margin predictors and 8 pathways were identified from molecular data analysis.

**Conclusion:**

The occurrence of positive margins after surgery was associated with various clinical factors, similar to the findings reported in earlier studies. In addition, we found that the PAM50 intrinsic subtype Luminal A has more chance of obtaining positive margins compared to Basal type. As the first effort to pursue molecular understanding of the margin status, a gene panel of 29 genes including 17 protein-coding genes was also identified for potential prediction of the margin status which needs to be validated using a larger sample set.

**Supplementary Information:**

The online version contains supplementary material available at 10.1007/s10549-023-07157-x.

## Introduction

Breast cancer remains a major health concern in the United States with an estimate of over 297,000 new cases for 2023 [1]. While survival rates have improved for breast cancer patients with advances in multimodality therapies, surgical resection with negative margins remains the standard of care for most patients. Most early-stage breast cancer patients are candidates for breast-conserving therapy (lumpectomy) or mastectomy for surgical resection given that multiple randomized controlled trials have demonstrated equivalent long-term survival outcomes [2–4]. Regardless of surgical strategy, margin status of the resected specimen remains one of the most important factors associated with recurrence after breast cancer surgery [5].

Positive surgical margins are defined as malignant cells identified at the edge of the resection specimen and have been associated with at least twofold increase in ipsilateral breast cancer recurrence [6, 7], higher distant recurrence rates, and shorter survival [8]. Further, patients with positive margins are candidates for re-excision of the concerned margin [6, 9] and these subsequent surgeries are associated with a significant burden to both the patients and healthcare system. While there have been several clinical factors associated with the risk of positive margins including higher stage, higher grade, non-ductal histology, HER2 amplification, and suspicion of multifocality, there is a paucity of data considering both clinical variables and genomic profiles associated with positive margins [10, 11].

In this study, we evaluated the clinical and pathologic factors associated with breast cancer surgical margins using the data for breast cancer (BRCA) from the public resource, The Cancer Genome Atlas (TCGA). In addition to clinical data analysis, exploration of molecular data was also performed in order to identify the genes potentially associated with positive margins.

## Materials and methods

### TCGA-BRCA data

The TCGA-BRCA patient data including clinical data and sample annotations were downloaded from the Genomic Data Commons (GDC) portal. The RNA-Seq data for the corresponding samples were also downloaded from GDC using the TCGA Biolinks R package [12]. The survival data were obtained from the TCGA Pan-Cancer study and integrated to the clinical data [13]. Since the number of the male patients was small and all of them had negative margin status, we excluded them to avoid the possibility of introducing additional bias. We also removed the redacted samples, and filtered cases using sample annotations. Finally 951 (75 positive and 876 negative margins) cases were retained for this study. The samples were categorized into positive and negative margin groups based on the margin status assigned after first tumor removal surgery.

The 951 sample cohort included primary tumors from patients diagnosed with breast cancer from 1988 to 2013 and had a median follow-up period of 2.2 years. Characteristics of the cohort were examined using frequency distributions and attributes with low numbers were grouped together as “Other.” The diagnosis age of the patients was in the range of 26 to 90 years and was grouped into three categories: old (60 + years), middle age (40–59 years) and young (< 40 years).

### Clinical data analysis

Clinicopathologic factors for margin prediction were evaluated using logistic regression models. Subsequently, Fisher’s exact test was performed to test the association of each factor with margins. The impact of each clinicopathologic feature on disease progression was evaluated using univariable and multivariable Cox proportional hazards regression with the recommended endpoint progression-free interval (PFI) [13]. The outcome of interest was time from date of diagnosis to local recurrence or distant metastasis or death from the disease whichever comes first. For margin status, Overall Survival (OS) was also estimated in addition to PFI. Significant factors from the univariable analysis were subjected to multivariable analysis to explore their effect on survival. In order to get a better understanding of the correlation of each significant factor in the multivariable model with survival, a bi-variable survival analysis was also performed. All analysis were carried out in R. All statistical tests were 2 sided, and *P* values ≤ 0.05 were considered significant.

### Molecular data analysis

A matched subset (*n* = 142) of the current TCGA dataset was selected for molecular analysis; all the cases with a positive margin that had tumor stage reported were included (*n* = 71) and the negative margin cases (*n* = 71) were selected by matching primarily on tumor stage and PAM50 subtype. Other features like race, age, and menopausal status were matched as much as possible. Principal component analysis (PCA) was performed to assess distribution of gene expression across PAM50 subtypes [14] and margin status. The RNA-Seq data analysis was performed using the package DESeq2 [15] with adjustment for PAM50 subtype and tumor stage. A 5% False Discovery Rate (FDR) and a fold change of 2 were established as significant criteria. The significant differentially expressed genes (DEGs) from DESeq2 were further subjected to LASSO regression [16] using caret package [17] in order to prevent multicollinearity and to extract the potential gene markers. A 10-fold cross-validation was performed to obtain the minimum lambda which was used in LASSO regression to predict the signature genes. Prediction models using Leave-One-Out Cross-Validation (LOOCV) [18] were performed to validate the gene signature. Additionally, pathway analysis was performed on the gene list from the DESeq2 result using the GSEA Preranked test tool against Hallmark gene set collection [19, 20].

## Results

### Positive margin is significantly associated with higher tumor stage and lumpectomy

The probability of attaining positive margins after surgery was observed to be significantly (*p* ≤ 0.05) associated with higher tumor stage, larger tumor size and chest wall involvement (T4), positive lymph nodes (N2, N3), and distant metastasis (M1), based on univariable logistic regression models and Fisher’s exact test (Table 1). The type of first surgery to remove tumor also influenced margin status with lumpectomy (as reference) having significantly higher chance of obtaining positive margins than mastectomy (Simple Mastectomy: *p* = 0.002, Odds Ratio (OR) = 0.30, Confidence Interval (CI) = 0.13 − 0.62; Modified Radical Mastectomy: *p* < 0.001, OR = 0.30, CI = 0.15 − 0.57). Among PAM50 subtypes, Luminal A subtype (as reference) was observed to be significantly contributing towards positive margin in the univariable regression model compared to the Basal-like (Basal) subtype (*p* = 0.05, OR = 0.44, CI = 0.18 − 0.94). Her2-enriched (Her2) subtype was associated with positive margins (OR = 1.39) although it was not significant (*p* = 0.397). The results of Fisher’s exact test were consistent with the logistic regression results except for PAM50 subtype which did not show any association with margin status.

**Table 1:** Summary of clinical characteristics of TCGA-BRCA data (*n* = 951) and their association with margin status

Clinical feature		*N*	Margin status no. (%)*		Fisher's test	Univariable logistic regression		
			Negative	Positive	*p*	*p*	OR^**^	95% CI^#^
Age group	Old (60 + years)	440	404 (46.1)	36 (48.0)	0.944	ref	ref	ref
	Middle Age (40–59)	449	414 (47.3)	35 (46.7)		0.832	0.95	0.58 − 1.54
	Young (< 40)	62	58 (6.6)	4 (5.3)		0.638	0.77	0.23 − 2.02
Menopausal status	Postmenopausal	624	575 (65.6)	49 (65.3)	0.269	ref	ref	ref
	Perimenopausal	37	35 (4.0)	2 (2.7)		0.590	0.67	0.11 − 2.29
	Premenopausal	209	192 (21.9)	17 (22.7)		0.896	1.04	0.57 − 1.81
	Indeterminate	27	22 (2.5)	5 (6.7)		0.058	2.67	0.86 − 6.84
	Not available	54	52 (5.9)	2 (2.7)		−	−	−
Tumor size (T)	T1	245	228 (26.0)	17 (22.7)	**0.014**	ref	ref	ref
	T2	559	521 (59.5)	38 (50.7)		0.942	0.98	0.55 − 1.81
	T3	119	105 (12.0)	14 (18.7)		0.126	1.79	0.84 − 3.76
	T4	26	20 (2.3)	6 (8.0)		**0.009**	4.02	1.33 − 10.95
	Not available	2	2 (0.2)	0 (0.0)		−	−	−
Lymph node status (N)	N0	458	434 (49.5)	24 (32.0)	**0.003**	ref	ref	ref
	N1	305	284 (32.4)	21 (28.0)		0.346	1.34	0.73 − 2.45
	N2	109	96 (11.0)	13 (17.3)		**0.013**	2.45	1.17 − 4.91
	N3	66	55 (6.3)	11 (14.7)		**0.001**	3.62	1.62 − 7.64
	Not available	13	7 (0.8)	6 (8.0)		−	−	−
Distant metastasis (M)	M0	796	737 (84.1)	59 (78.7)	** < 0.001**	ref	ref	ref
	M1	14	6 (0.7)	8 (10.7)		** < 0.001**	16.66	5.61 − 52.11
	Not Available	141	133 (15.2)	8 (10.7)		−	−	−
AJCC^a^ stage	Stage I	161	153 (17.5)	8 (10.7)	** < 0.001**	ref	ref	ref
	Stage II	544	515 (58.8)	29 (38.7)		0.857	1.08	0.50 − 2.57
	Stage III	217	191 (21.8)	26 (34.7)		**0.023**	2.60	1.20 − 6.30
	Stage IV	12	4 (0.5)	8 (10.7)		** < 0.001**	38.24	10.02 − 171.69
	Not available	17	13 (1.5)	4 (5.3)		−	−	−
PAM50	Luminal A	488	445 (50.8)	43 (57.3)	0.112	ref	ref	ref
	Luminal B	182	170 (19.4)	12 (16.0)		0.354	0.73	0.36 − 1.38
	Basal	171	164 (18.7)	7 (9.3)		**0.050**	0.44	0.18 − 0.94
	Her2	76	67 (7.6)	9 (12.0)		0.397	1.39	0.61 − 2.86
	Normal	34	30 (3.4)	4 (5.3)		0.562	1.38	0.40 − 3.70
Race	White	659	612 (69.9)	47 (62.7)	0.170	ref	ref	ref
	African American	157	141 (16.1)	16 (21.3)		0.199	1.48	0.79 − 2.62
	Other^e^	57	56 (6.4)	1 (1.3)		0.153	0.23	0.01 − 1.10
	Not available	78	67 (7.6)	11 (14.7)		−	−	−
Type of first surgery	Lumpectomy	222	189 (21.6)	33 (44.0)	** < 0.001**	ref	ref	ref
	Simple mastectomy	181	172 (19.6)	9 (12.0)		**0.002**	0.30	0.13 − 0.62
	Modified radical Mastectomy	281	267 (30.5)	14 (18.7)		** < 0.001**	0.30	0.15 − 0.57
	Other^f^	231	212 (24.2)	19 (25.3)		**0.029**	0.51	0.28 − 0.92
	Not available	36	36 (4.1)	0 (0.0)		−	−	−
ER^b^ status	Negative	215	203 (23.2)	12 (16.0)	0.119	ref	ref	ref
	Positive	697	634 (72.4)	63 (84.0)		0.110	1.68	0.92 − 3.33
	Not available	39	39 (4.5)	0 (0.0)		−	−	−
PR^c^ Status	Negative	311	288 (32.9)	23 (30.7)	0.528	ref	ref	ref
	Positive	598	546 (62.3)	52 (69.3)		0.500	1.19	0.72 − 2.02
	Not available	42	42 (4.8)	0 (0.0)		−	−	−
HER2^d^ status	Negative	498	463 (52.9)	35 (46.7)	0.853	ref	ref	ref
	Positive	140	130 (14.8)	10 (13.3)		0.963	1.02	0.47 − 2.04
	Equivocal	157	144 (16.4)	13 (17.3)		0.600	1.19	0.59 − 2.27
	Not available	156	139 (15.9)	17 (22.7)		−	−	−
Histology	Ductal	677	629 (71.8)	48 (64.0)	0.333	ref	ref	ref
	Lobular	186	167 (19.1)	19 (25.3)		0.161	1.49	0.84 − 2.57
	Mixed	23	20 (2.3)	3 (4.0)		0.289	1.97	0.45 − 5.99
	Mucinous	16	14 (1.6)	2 (2.7)		0.416	1.87	0.29 − 6.96
	Others^g^	48	45 (5.1)	3 (4.0)		0.826	0.87	0.21 − 2.51
	Not available	1	1 (0.1)	0 (0.0)		−	−	−

*Number of cases (percentage within each margin group)

**Odds ratio

^#^Confidence interval of odds ratio

^a^American joint committee on cancer

^b^Estrogen Receptor

^c^Progestrone Receptor

^d^Human Epidermal Growth Factor Receptor 2

^e^Asians and American Indians

^f^Other types of surgeries like partial mastectomy, Patey's surgery, excision with needle wire localization etc.

^g^Medullary carcinoma, Metaplastic carcinoma and other types of histology

Bold lettering denotes p value ≤ 0.05

The significant factors in the univariable regression model (Stage, PAM50, TNM: T = Tumor size, N = Lymph Node status, M = Metastasis, Type of first surgery) were used in the multivariable model with margin status as response variable. Tumor stage, size, and lymph node status, which were highly significant in the univariable model, were no longer significant in the multivariable model (Supplementary Table S1). Further evaluation using various multivariable models proved that Stage and TNM were confounding (Supplementary Table S2); hence, only Stage was used in the final multivariable model (Table 2). The final regression model, in agreement with the univariable model, showed that patients diagnosed at higher tumor stage (Stage III: *p* < 0.001, OR = 4.85, CI = 2.09 − 12.41; Stage IV: *p* < 0.001, OR = 80.83, CI = 18.65 − 411.45) were significantly associated with positive margins. Similarly, in case of type of surgery for tumor removal, the multivariable regression model reemphasized that lumpectomy (as reference) was significantly associated with positive margin compared to simple mastectomy (*p* = 0.002, OR = 0.27, CI = 0.12 − 0.59) and modified radical mastectomy (*p* < 0.001, OR = 0.17, CI = 0.08 − 0.35). For the PAM50 subtypes, Luminal A (as reference) was significantly associated with positive margins compared to basal subtype (*p* = 0.042, OR = 0.41, CI = 0.16 − 0.91).

**Table 2:** Multivariable logistic regression analysis on the association of margin status with significant clinical features from univariable analysis

Clinical feature		Multivariable logistic regression		
		*p*	OR^*^	95% CI^**^
AJCC stage	Stage I	ref	ref	ref
	Stage II	0.343	1.49	0.68 − 3.64
	Stage III	** < 0.001**	4.85	2.09 − 12.41
	Stage IV	** < 0.001**	80.83	18.65 − 411.45
PAM50	Luminal A	ref	ref	ref
	Luminal B	0.109	0.53	0.24 − 1.11
	Basal	**0.042**	0.41	0.16 − 0.91
	Her2	0.980	1.01	0.41 − 2.26
	Normal	0.989	1.01	0.24 − 3.19
Type of first surgery	Lumpectomy	ref	ref	ref
	Simple mastectomy	**0.002**	0.27	0.12 − 0.59
	Modified radical mastectomy	** < 0.001**	0.17	0.08 − 0.35
	Other^a^	**0.005**	0.38	0.19 − 0.73

*Odds ratio

**Confidence interval for odds ratio

^a^Surgery other than lumpectomy or simple/radical mastectomy. For e.g. Needle wire excision

Bold lettering denotes *p* value ≤ 0.05

### Effect of margin status and other factors on disease progression

**Fig. 1:**
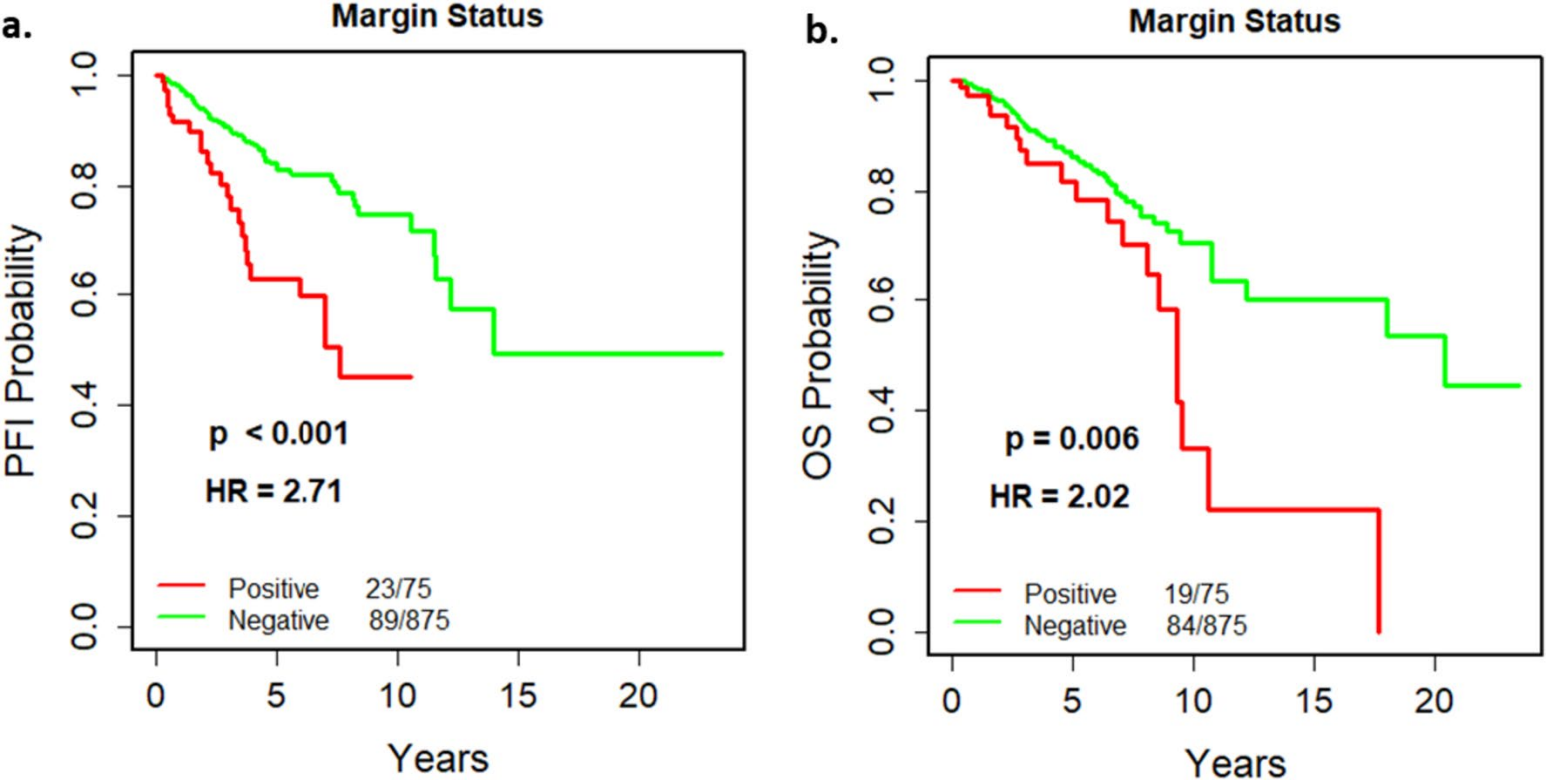
The Kaplan–Meier (K–M) curves for cumulative survival in years for margin status for two end points: progression-free interval (PFI) (**a**) and overall survival (OS) (**b**). *P* value, Hazard ratio (HR), and the number of events ‘/’ number of cases are given in the legends of plots

Of the 951 cases included in our study, one was excluded from survival analysis due to missing follow-up information. The univariable survival models using 950 cases for margin status showed that positive margins were significantly associated with worse survival with both PFI (*p* < 0.001) and OS (*p* = 0.006) as endpoints (Fig. 1). In addition to margin status, stage, TNM, PAM50 subtype, and hormone receptor (Estrogen Receptor (ER), Progesterone Receptor (PR)) status were significantly associated with disease progression (Table 3). While examining the survival models based on histology, mucinous carcinoma was found to have significant survival difference compared to ductal carcinoma (Table 3). However, as the sample size (*n* =15) and the number of events (*n* =3) were low for mucinous carcinoma (Supplementary Fig.S1), these results were regarded as unreliable. It is worth noting that the type of first surgery, though significantly associated with margin status, does not significantly impact survival.

**Table 3:** Univariable survival analysis to assess the effect of each clinicopathologic factor on disease progression (Progression Free Interval, PFI)

Clinical feature		Univariable survival analysis		
		*p*	HR^*^	95% CI^**^
Margin status	Negative	ref	ref	ref
	Positive	** < 0.001**	2.71	1.70 − 4.30
Age group	60 + years	ref	ref	ref
	40–59 years	0.176	0.76	0.51 − 1.13
	< 40 years	0.379	1.33	0.71 − 2.50
Menopausal status	Postmenopausal	ref	ref	ref
	Perimenopausal	0.320	0.49	0.12 − 2.00
	Premenopausal	0.985	1.00	0.64 − 1.56
	Indeterminate	0.100	1.71	0.90 − 3.25
Tumor size (T)	T1	ref	ref	ref
	T2	0.150	1.43	0.88 − 2.34
	T3	**0.048**	1.88	1.00 − 3.50
	T4	** < 0.001**	7.64	3.73 − 15.64
	Not available	−	−	−
Lymph node status (N)	N0	ref	ref	ref
	N1	0.069	1.55	0.97 − 2.47
	N2	**0.001**	2.66	1.51 − 4.71
	N3	** < 0.001**	5.25	2.91 − 9.44
	Not available	−	−	−
Distant metastasis (M)	M0	ref	ref	ref
	M1	** < 0.001**	8.96	4.76 − 16.87
	Not available	−	−	−
AJCC stage	Stage I	ref	ref	ref
	Stage II	0.478	1.25	0.67 − 2.33
	Stage III	** < 0.001**	3.17	1.69 − 5.96
	Stage IV	** < 0.001**	13.24	5.64 − 31.07
PAM50	Luminal A	ref	ref	ref
	Luminal B	0.451	1.23	0.72 − 2.09
	Basal	**0.033**	1.67	1.04 − 2.68
	Her2	**0.015**	2.15	1.16 − 3.99
	Normal	0.451	1.43	0.57 − 3.59
Race	White	ref	ref	ref
	African American	0.313	1.27	0.80 − 2.02
	Other	0.235	1.68	0.68 − 4.19
First surgery	Lumpectomy	ref	ref	ref
	Simple mastectomy	0.527	0.83	0.46 − 1.49
	Modified radical mastectomy	0.361	1.26	0.77 − 2.05
	Other	0.372	0.78	0.44 − 1.36
ER status	Negative	ref	ref	ref
	Positive	**0.006**	0.58	0.39 − 0.86
PR status	Negative	ref	ref	ref
	Positive	**0.001**	0.51	0.35 − 0.75
HER2 status	Negative	ref	ref	ref
	Positive	0.394	1.30	0.71 − 2.39
	Equivocal	0.304	1.32	0.78 − 2.22
Histology	Ductal	ref	ref	ref
	Lobular	0.951	0.98	0.60 − 1.61
	Mixed	0.917	1.05	0.39 − 2.89
	Mucinous	**0.028**	3.67	1.15 − 11.70
	Others	0.350	1.42	0.68 − 2.94

*Hazard ratio

**Confidence interval for hazard ratio

Bold lettering denotes *p* value ≤ 0.05

In order to assess the combined effect of margin status and other factors that were significant in the univariable model on survival, multivariable survival analysis was performed. TNM, though significant in the univariable model, was excluded in the multivariable models since tumor stage is derived from TNM and the inclusion of both features in the same model was observed to be confounding in the previous logistic regression model. Surprisingly, PAM50 and ER status were not significant in this model (Supplementary Table S3). Further exploration using different multivariable models (Supplementary Tables S4–S5) indicated that hormone receptor status and PAM50 were confounding to each other; hence only PAM50 was retained in the final model (Table 4). Higher tumor stages (III and IV), and the Basal and Her2 subtypes were significant (*p* ≤ 0.05) in contribution to disease progression in the final model, while margin status was not significant (*p* = 0.135, HR = 1.54, CI = 0.88 − 2.70). The bi-variable survival models (Table 5) demonstrated that margin status remained highly significant when PAM50 or either of the hormone receptor (ER/PR) status was added to the model whereas in the model with tumor stage, margin status was only close to significance (*p* = 0.067).

**Table 4:** Final Cox proportional hazards regression model for multivariable survival analysis

Clinical feature		Multivariable survival analysis		
		*p*	HR^*^	95% CI^**^
Margin status	Negative	ref	ref	ref
	Positive	0.135	1.54	0.88 − 2.70
AJCC stage	Stage I	ref	ref	ref
	Stage II	0.771	1.10	0.59 − 2.06
	Stage III	**0.001**	3.05	1.59 − 5.88
	Stage IV	** < 0.001**	11.80	4.50 − 30.94
PAM50	LumA	ref	ref	ref
	LumB	0.803	1.08	0.59 − 1.97
	Basal	**0.002**	2.42	1.40 − 4.19
	Her2	**0.032**	2.11	1.06 − 4.19
	Normal	0.464	1.42	0.56 − 3.61
Histology	Ductal	ref	ref	ref
	Lobular	0.478	1.24	0.69 − 2.22
	Mixed	0.734	0.81	0.24 − 2.74
	Mucinous	**0.008**	5.09	1.53 − 16.91
	Others	0.211	1.66	0.75 − 3.67

*Hazard ratio

**Confidence interval for hazard ratio

Bold lettering denotes *p* value ≤ 0.05

**Table 5:** Assessment of impact of each significant factor from univariable models on margin status using bi-variable survival analysis with Progression Free Interval (PFI) as endpoint

Margin status^#^			2nd variable				
*p*	HR^*^	95% CI^**^			*p*	HR	95% CI
0.067	1.66	0.96 − 2.86	**AJCC Stage**	Stage I	ref	ref	ref
				Stage II	0.482	1.25	0.67 − 2.32
				Stage III	**0.001**	2.98	1.58 − 5.61
				Stage IV	** < 0.001**	9.56	3.78 − 24.19
** < 0.001**	2.83	1.77 − 4.53	**PAM50**	Luminal A	ref	ref	ref
				Luminal B	0.334	1.30	0.76 − 2.21
				Basal	**0.011**	1.86	1.15 − 3.00
				Her2	**0.032**	1.97	1.06 − 3.65
				Normal	0.512	1.36	0.54 − 3.43
** < 0.001**	3.04	1.90 − 4.85	**ER**	Negative	ref	ref	ref
				Positive	**0.002**	0.52	0.35 − 0.78
** < 0.001**	2.98	1.87 − 4.75	**PR**	Negative	ref	ref	ref
				Positive	** < 0.001**	0.48	0.33 − 0.71

*Hazard ratio

**Confidence Interval for hazard ratio

^#^Margin status was used as the first variable in all bi-variable models with negative margin kept as reference

Bold lettering denotes *p* value ≤ 0.05

### Association of gene expression with margin status identified 29 DEGs

**Fig. 2:**
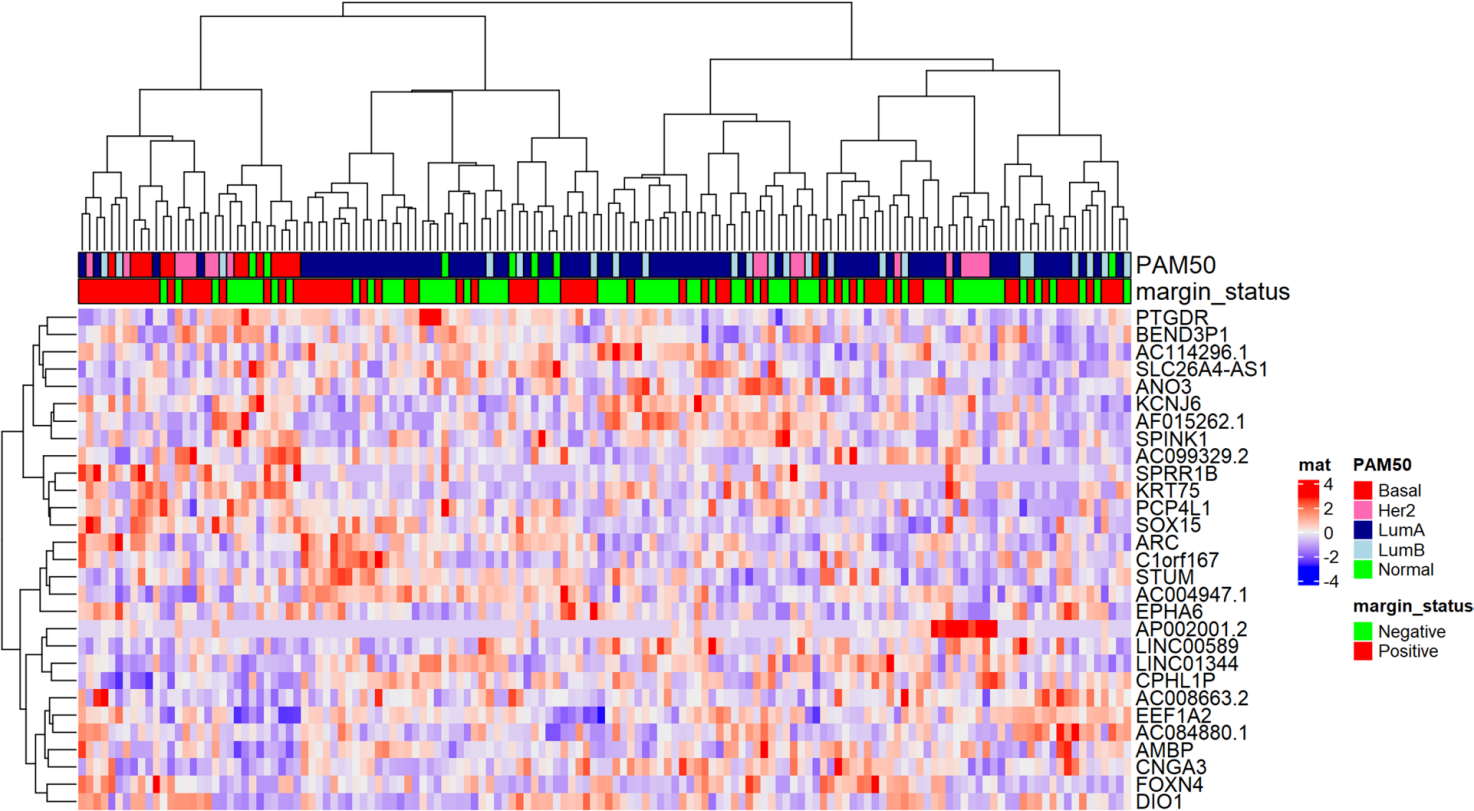
Unsupervised clustering for 29 significant genes derived using LASSO regression from Deseq2 analysis for TCGA RNA-Seq data

To address the sample imbalance between positive and negative margins, a matched dataset (*n* = 142; Supplementary Table S6) was extracted from our cohort to perform unbiased molecular analyses. Principal component analysis (PCA) of matched samples using 2000 highly varying genes did not clearly cluster the samples by margin status but clustered them instead by PAM50 subtypes (Supplementary Fig. S2). Differential expression analysis between positive and negative margin cases discovered 53 upregulated and 50 downregulated DEGs and the subsequent LASSO regression selected 29 DEGs for the prediction of margin (Supplementary Table S7). The unsupervised clustering for these 29 genes demonstrated largely subtype-driven clusters (Fig. 2). We also observed two main level clusters that have different positive margin enrichment (~ 59% for left cluster, ~ 41% for right cluster, Fisher’s exact *p* value = 0.044). This show the genes to some degree can separate the positive margin from negative margin. Leave-One-Out Cross-Validation (LOOCV)-based prediction models with the 29 genes showed an accuracy of 0.7.

Among the 29 genes, 16 were upregulated and 13 were downregulated in positive margin cases. It included 17 protein-coding genes, 4 pseudogenes (*AC084880.1, BEND3P1, CPHL1P, AP002001.2*), and 8 long non-coding RNA (LncRNA) genes (*AC004947.1, AC008663.2, AC099329.2, LINC01344, SLC26A4-AS1, AF015262.1, AC114296.1, LINC00589*).

Pathway analysis identified 8 differentially expressed pathways (Table 6) between positive and negative margin cases. The 7 upregulated pathways include three cell proliferation associated pathways (E2F_TARGETS, G2M_CHECKPOINT, MYC_TARGETS_V1); two cell signaling-related pathways (ESTROGEN_RESPONSE_LATE, ESTROGEN_RESPONSE_EARLY); and two immune-related pathways (INTERFERON_ALPHA_RESPONSE, TNF_SIGNALING_VIA_NFKB). The only downregulated pathway was associated with progression and metastasis (EPITHELIAL_MESENCHYMAL_TRANSITION).

**Table 6:** Significant pathways observed in TCGA RNA-Seq data (*n* = 142) using GSEA Preranked test

#	NAME	NES	FDR	Up/down
1	E2F_TARGETS	1.965	0.002	↑
2	ESTROGEN_RESPONSE_LATE	1.875	0.002	↑
3	ESTROGEN_RESPONSE_EARLY	1.824	0.002	↑
4	TNFA_SIGNALING_VIA_NFKB	1.720	0.005	↑
5	INTERFERON_ALPHA_RESPONSE	1.698	0.005	↑
6	G2M_CHECKPOINT	1.584	0.020	↑
7	MYC_TARGETS_V1	1.553	0.023	↑
8	EPITHELIAL_MESENCHYMAL_TRANSITION	− 1.836	0.003	↓

## Discussion

### Characterization study to determine effects of factors on margin status

Here, we performed the analysis of both clinicopathologic and molecular factors with the occurrence of positive margins in breast cancer. The incidence rate of positive margins (7.8%; 75/951) in TCGA-BRCA was comparable to other studies [21]. We observed that the risk of positive margins increases with higher tumor stage, larger tumor size, positive lymph nodes, and presence of distant metastasis consistent with prior studies [21–24]. Conversely, age which has been previously reported to be associated with margin status was not significant in our analysis [22]. This discordance could be attributed to the low number of young patients in the TCGA-BRCA cohort.

At the molecular level, our study demonstrated that immunohistochemistry (IHC) markers ER, PR, and HER2 did not impact margin status, and this is in concordance with the findings described by Horattas et al. [25]. Evaluation of PAM50 intrinsic subtypes in our study, however, demonstrated that Luminal A subtype had a higher risk of positive margins compared with Basal subtype. There is a paucity of literature considering the impact of PAM50 subtypes on margin status, and this novel finding appears to be counter intuitive, given that Basal subtype is associated with higher recurrence rates [26]. The higher incidence of positive margins in the Luminal A subtype may be attributed to morphologic characteristics noted in radiomic studies which attribute spiculated features more commonly to Luminal subtypes and circumscribed features more commonly to Basal subtypes [27].

The survival analysis demonstrated that positive margin status, larger tumor size, positive lymph nodes, distant metastasis, hormone receptor status (ER-negative, PR-negative), higher tumor stage, and two of the PAM50 subtypes (Basal and Her2) significantly contributed to disease progression. This conclusion agrees with previous studies including those using TCGA data, even though margin status was not evaluated in prior TCGA-BRCA data studies [13, 28, 29]. The bi-variable survival models indicated that margin status acted independently from PAM50 or hormone receptor status. It also suggested that margin status might be a surrogate to tumor stage.

### Mastectomy does not guarantee negative margins

Per current guidelines, mastectomy is typically indicated for breast cancer patients with larger tumor size relative to breast size, inflammatory breast cancer, multicentric disease, and patient preference as well as in patients with a contraindication to breast-conserving therapy. Patients may prefer a mastectomy over a lumpectomy for a variety of reasons including a decreased risk of positive margins. Our findings are consistent with the literature in regard to the higher risk of positive margins in patient undergoing lumpectomy [6]. Interestingly, Hewitt, et al. reported that in patients with large invasive lobular carcinoma (ILC) tumors, mastectomy fails to obtain clear margins [30]. Out of the 23 patients with positive margins after mastectomy (simple/radical) in our cohort, 15 of them had invasive ductal carcinoma (IDC) and 7 had ILC. It is important to note that, 21 out of these 23 tumors belonged to the higher stage group (Stage III or IV). This again emphasizes higher stage patients has higher chance of undergoing re-excisions irrespective of type of first surgery or histology.

### Biomarkers for margin status

To our knowledge, this is the first study to evaluate the impact of gene expression on margin status. The 29 genes obtained after molecular data analysis included many well-known cancer markers. The 12 protein-coding genes upregulated in positive margin group included several well-known tumor markers for breast cancer (*EEF1A2, EPHA6, FOXN4, SOX15*) [31–35]. In addition, there were genes that were reported in other cancers but were not much explored in breast cancer. The low expression of Alpha-1-Microglobulin/Bikunin Precursor (*AMBP*) has been reported to increase tumor progression in prostate cancer [36] and oral squamous carcinoma [37], but is relatively understudied in breast cancer. A study exploring expression of this gene across different cancers reported it to be downregulated in breast cancer [38]. However, this gene was found to be overexpressed in our positive margin cohort. Similarly, Iodothyronine Deiodinase 1 (*DIO1*), a gene involved in the activation and inactivation of thyroid hormone whose low expression is said to promote tumor progression was also upregulated in patients with positive margins [39]. These findings with AMBP and DIO1 genes may merit further studies. In addition, we also identified 6 upregulated genes not typically associated with cancer (ARC, C1orf167, CNGA3, KRT75, SPRR1B, STUM).

There were 5 downregulated protein-coding gene identified in the positive margin group including *SPINK1*, a well-known tumor marker [40, 41]. Potassium Inwardly Rectifying Channel Subfamily J Member 6 (*KCNJ6*) was also downregulated in positive margin cases. Potassium channel-driven signaling is known to regulate metastasis in triple negative cancer [42]. Anoctamin 3 (*ANO3*) was another downregulated genes whose paralogue *ANO1* is a known cancer marker for head and neck squamous carcinoma [43]. The Prostaglandin D2 Receptor (*PTGDR*) also called *PGD2* has been associated with different type of cancers [44] even though its role in breast cancer has not been well described. However, it has been reported that high expression of *PGD2* resulted in reduced tumor proliferation [45]. This might explain the reason for *PGD2* being significantly downregulated in positive margin cases in our cohort. There is limited knowledge of *PCP4L1* in malignancies.

The unsupervised clustering of these 29 genes grouped the samples primarily by subtype, although different enrichment of margin positive samples was observed from the two main clusters. A larger sample size of patients with positive margins is needed to validate these genes as predictors of margin status. Since positive margin is a strong indicator for breast cancer recurrence, these genes in turn could be considered as potential markers of recurrence.

Furthermore, the pathway analysis revealed prominent pathways like MYC_TARGETS, E2F_TARGETS that have been reported by previous studies to be associated with breast cancer recurrence (Table 6) [46]. Estrogen response-related pathways were upregulated in margin positive samples further emphasizing our previous observation of Luminal A having higher chance of positive margin compared to basal subtype (Table 2).

## Conclusion

The clinical data analysis results using TCGA-BRCA data show that higher stage, larger tumor size, positive lymph nodes, presence of distant metastasis, and Luminal A subtypes have higher chance of obtaining positive margins after first surgery. We also observed that mastectomy for tumor removal reduced chance of positive margins compared to lumpectomy. This is in agreement with the previously reported studies. However, we also found that margin status likely was a surrogate to tumor stage and, hence, patients diagnosed at higher stage, regardless of type of surgery had higher chance of obtaining positive margins. Additionally, we also observed that patients belonging to Luminal A intrinsic subtype had higher chance of obtaining positive margins compared to Basal subtype. Based on these findings, patients with Luminal A or higher stage tumors should be counseled on their increased risk of positive margins. Clinical indications for wider margin resection for these patients would require further rigorous examination in a clinical trial prior to definitely altering guidelines. We also identified 29 genes and 8 pathways significantly differential expressed between positive and negative margins. These 29 genes, some of which had not been reported to be associated with breast cancer previously, could serve as potential predictors of margin status. However, additional studies need to be performed on a larger sample size to validate these findings. On-going studies to further identify risk factors associated with positive margins will help physicians in determining treatment strategy and counseling their patients.

### Supplementary Information

Below is the link to the electronic supplementary material.

Supplementary file1 (DOCX 337 KB)

## Data Availability

The dataset analyzed in the current study is from and available in the TCGA repository, https://portal.gdc.cancer.gov/. All results generated during this study are included in this published article and its supplementary information file.
