# Supplementary material for: Association of clinicopathologic and molecular factors with the occurrence of positive margins in breast cancer
Source: Breast Cancer Res Treat. 2023 Dec 1;204(1):15–26. doi: 10.1007/s10549-023-07157-x (PMC10805852; doi:10.1007/s10549-023-07157-x)
Supplement: Supplementary file 1 — Supplementary file1 (DOCX 337 KB) [file 10549_2023_7157_MOESM1_ESM.docx]

# SUPPLEMENTARY INFORMATION

Supplementary Table S1. Multivariable logistic regression analysis on the association of margin status with all the significant factors from univariable analysis.

| **Clinical Feature** |  | **Multivariable Logistic Regression** | | |
| --- | --- | --- | --- | --- |
|  |  | **p** | **OR^*^** | **95% CI^**^** |
| Tumor Size  (T) | T1 | ref | ref | ref |
|  | T2 | 0.617 | 1.32 | 0.48 − 4.29 |
|  | T3 | 0.163 | 2.53 | 0.71 − 9.93 |
|  | T4 | 0.288 | 2.73 | 0.41 − 17.52 |
|  |  |  |  |  |
| Lymph Node Status  (N) | N0 | ref | ref | ref |
|  | N1 | 0.884 | 1.06 | 0.46 − 2.42 |
|  | N2 | 0.346 | 2.15 | 0.44 − 11.06 |
|  | N3 | 0.393 | 1.98 | 0.42 − 9.85 |
|  |  |  |  |  |
| Distant Metastasis  (M) | M0 | ref | ref | ref |
|  | M1 | **0.037** | 15.39 | 1.16 − 210.79 |
|  |  |  |  |  |
| AJCC Stage | Stage I | ref | ref | ref |
|  | Stage II | 0.891 | 0.91 | 0.22 − 3.53 |
|  | Stage III | 0.749 | 1.40 | 0.17 − 10.62 |
|  | Stage IV | NA | NA | NA |
|  |  |  |  |  |
| PAM50 | Luminal A | ref | ref | ref |
|  | Luminal B | 0.111 | 0.50 | 0.21 − 1.12 |
|  | Basal | 0.104 | 0.48 | 0.18 − 1.10 |
|  | Her2 | 0.844 | 1.09 | 0.42 − 2.56 |
|  | Normal | 0.748 | 0.78 | 0.12 − 3.01 |
|  |  |  |  |  |
| Type of First Surgery | Lumpectomy | ref | ref | ref |
|  | Simple Mastectomy | **0.010** | 0.33 | 0.14 − 0.74 |
|  | Modified Radical Mastectomy | **< 0.001** | 0.15 | 0.06 − 0.35 |
|  | Other | **0.002** | 0.31 | 0.14 − 0.63 |

^*^Odds Ratio

^**^Confidence Interval for Odds Ratio

Bold lettering denotes p value ≤ 0.05

Supplementary Table S2: Multivariable logistic regression analysis on the association of margin status with significant features from univariable analysis including TNM instead of Stage.

| **Clinical Feature** |  | **Multivariable Logistic Regression** | | |
| --- | --- | --- | --- | --- |
|  |  | **p** | **OR^*^** | **95% CI^**^** |
| Tumor Size  (T) | T1 | ref | ref | ref |
|  | T2 | 0.490 | 1.28 | 0.64 − 2.69 |
|  | T3 | **0.031** | 2.79 | 1.09 − 7.12 |
|  | T4 | 0.123 | 3.20 | 0.66 − 13.16 |
|  |  |  |  |  |
| Lymph Node Status  (N) | N0 | ref | ref | ref |
|  | N1 | 0.634 | 1.18 | 0.59 − 2.31 |
|  | N2 | **0.008** | 3.07 | 1.30 − 6.99 |
|  | N3 | **0.038** | 2.83 | 1.01 − 7.38 |
|  |  |  |  |  |
| Distant Metastasis  (M) | M0 | ref | ref | ref |
|  | M1 | **0.002** | 8.60 | 2.15 − 33.88 |
|  |  |  |  |  |
| PAM50 | Luminal A | ref | ref | ref |
|  | Luminal B | 0.116 | 0.51 | 0.21 − 1.12 |
|  | Basal | 0.078 | 0.46 | 0.18 − 1.03 |
|  | Her2 | 0.897 | 1.06 | 0.42 − 2.45 |
|  | Normal | 0.736 | 0.77 | 0.11 − 2.97 |
|  |  |  |  |  |
| Type of First Surgery | Lumpectomy | ref | ref | ref |
|  | Simple Mastectomy | **0.007** | 0.32 | 0.13 − 0.71 |
|  | Modified Radical Mastectomy | **< 0.001** | 0.14 | 0.06 − 0.32 |
|  | Other | **0.001** | 0.29 | 0.14 − 0.59 |

^*^Odds Ratio

^**^Confidence Interval for Odds Ratio

Bold lettering denotes p value ≤ 0.05


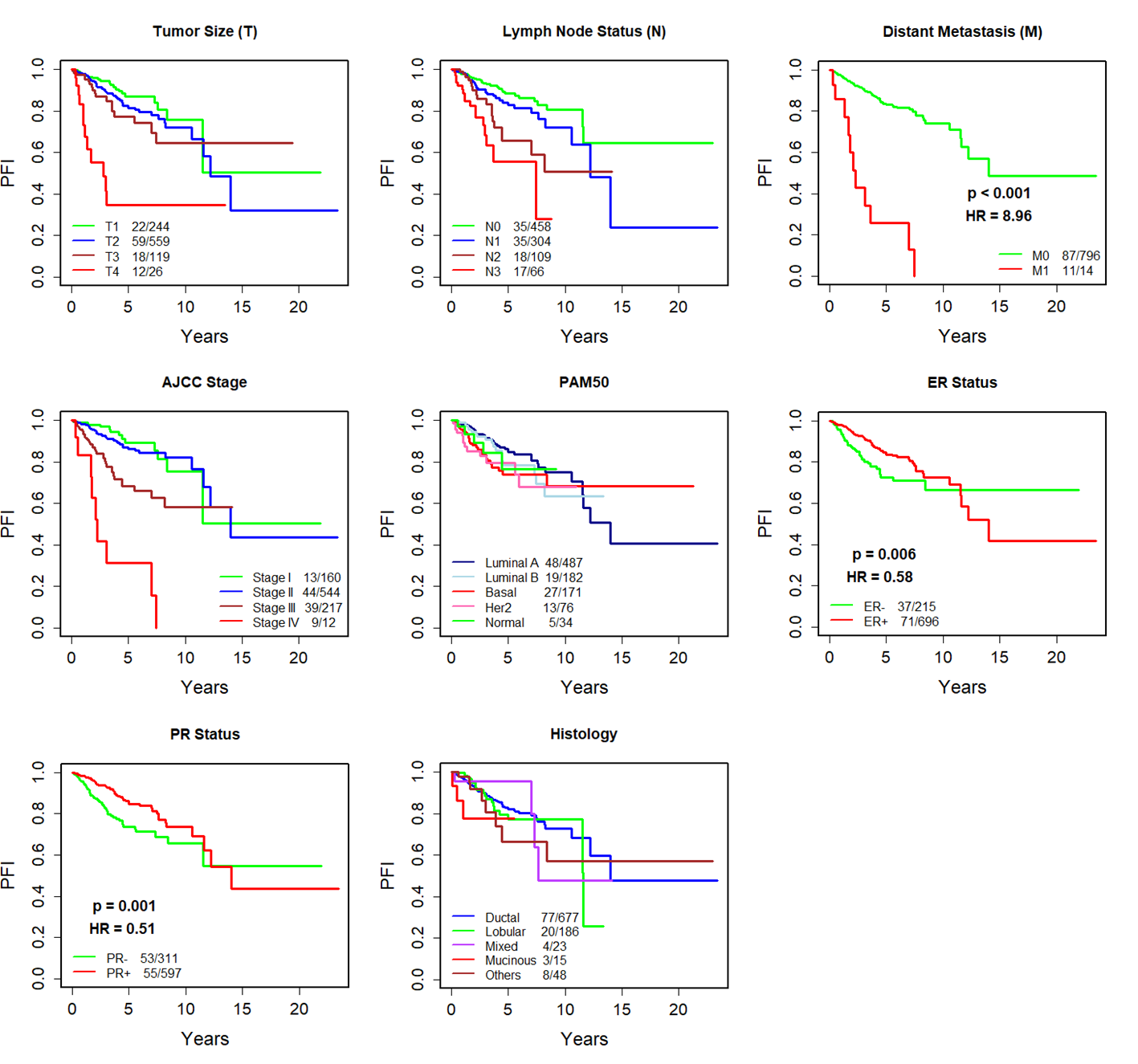


Supplementary **Fig.S1**: The Kaplan–Meier curves for cumulative survival in years for our cohorts as defined by Tumor Size, Lymph Node Status, Distant Metastasis, AJCC Stage, PAM50, ER status, PR status, and histology for a recommended end point in the TCGA-BRCA cohort: progression-free interval (PFI). P-value (p), Hazard ratio (HR) and the number of events ‘/’ number of cases are given in the legends of plots.

Supplementary Table S3. Multivariable survival model with Progression Free Interval (PFI) as the endpoint and includes the significant features identified in univariable survival model.

| **Clinical Feature** | | **Multivariable Survival Analysis** | | |
| --- | --- | --- | --- | --- |
|  |  | **p** | **HR^*^** | **95% CI^**^** |
| Margin Status | Negative | ref | ref | ref |
|  | Positive | 0.105 | 1.61 | 0.91 − 2.87 |
|  |  |  |  |  |
| AJCC Stage | Stage I | ref | ref | ref |
|  | Stage II | 0.613 | 1.18 | 0.62 − 2.23 |
|  | Stage III | **< 0.001** | 3.29 | 1.70 − 6.36 |
|  | Stage IV | **< 0.001** | 13.81 | 5.14 − 37.12 |
|  |  |  |  |  |
| PAM50 | LumA | ref | ref | ref |
|  | LumB | 0.637 | 0.86 | 0.45 − 1.63 |
|  | Basal | 0.898 | 1.05 | 0.48 − 2.30 |
|  | Her2 | 0.897 | 1.06 | 0.45 − 2.48 |
|  | Normal | 0.816 | 1.12 | 0.43 − 2.94 |
|  |  |  |  |  |
| ER | Negative | ref | ref | ref |
|  | Positive | 0.440 | 0.77 | 0.39 − 1.50 |
|  |  |  |  |  |
| PR | Negative | ref | ref | ref |
|  | Positive | **0.023** | 0.49 | 0.26 − 0.90 |
|  |  |  |  |  |
| Histology | Ductal | ref | ref | ref |
|  | Lobular | 0.679 | 1.14 | 0.61 − 2.10 |
|  | Mixed | 0.984 | 0.99 | 0.29 − 3.36 |
|  | Mucinous | **0.003** | 6.21 | 1.83 − 21.04 |
|  | Others | 0.180 | 1.72 | 0.78 − 3.81 |

^*^Hazard Ratio, ^**^Confidence Interval for hazard ratio

Bold lettering denotes p value ≤ 0.05

Supplementary Table S4: Multivariable survival analysis model without PAM50

| **Clinical Feature** |  | **Multivariable Survival Analysis** | | |
| --- | --- | --- | --- | --- |
|  |  | **p** | **HR^*^** | **95% CI^**^** |
|  |  |  |  |  |
| Margin Status | Negative | ref | ref | ref |
|  | Positive | 0.069 | 1.67 | 0.96 − 2.92 |
|  |  |  |  |  |
| AJCC Stage | Stage I | ref | ref | ref |
|  | Stage II | 0.599 | 1.18 | 0.63 − 2.21 |
|  | Stage III | **< 0.001** | 3.23 | 1.69 − 6.17 |
|  | Stage IV | **< 0.001** | 13.46 | 5.09 − 35.57 |
|  |  |  |  |  |
| ER | Negative | ref | ref | ref |
|  | Positive | 0.281 | 0.72 | 0.39 − 1.31 |
|  |  |  |  |  |
| PR | Negative | ref | ref | ref |
|  | Positive | **0.012** | 0.48 | 0.27 − 0.85 |
|  |  |  |  |  |
| Histology | Ductal | ref | ref | ref |
|  | Lobular | 0.583 | 1.18 | 0.66 − 2.10 |
|  | Mixed | 0.979 | 1.02 | 0.30 − 3.41 |
|  | Mucinous | **0.003** | 6.19 | 1.84 − 20.83 |
|  | Others | 0.164 | 1.76 | 0.79 − 3.88 |

^*^Hazard Ratio, ^**^Confidence Interval for hazard ratio

Bold lettering denotes p value ≤ 0.05

Supplementary Table S5: Multivariable survival analysis model without PAM50 & Progesterone receptor (PR) status.

| **Clinical Feature** |  | **Multivariable Survival Analysis** | | |
| --- | --- | --- | --- | --- |
|  |  | **p** | **HR^*^** | **95% CI^**^** |
|  |  |  |  |  |
| Margin Status | Negative | ref | ref | ref |
|  | Positive | 0.065 | 1.69 | 0.97 − 2.95 |
|  |  |  |  |  |
| AJCC Stage | Stage I | ref | ref | ref |
|  | Stage II | 0.636 | 1.16 | 0.62 − 2.18 |
|  | Stage III | **< 0.001** | 3.21 | 1.68 − 6.15 |
|  | Stage IV | **< 0.001** | 13.25 | 5.03 − 34.90 |
|  |  |  |  |  |
| ER | Negative | ref | ref | ref |
|  | Positive | **< 0.001** | 0.42 | 0.26 − 0.66 |
|  |  |  |  |  |
| Histology | Ductal | ref | ref | ref |
|  | Lobular | 0.811 | 1.07 | 0.60 − 1.90 |
|  | Mixed | 0.880 | 0.91 | 0.27 − 3.04 |
|  | Mucinous | **0.007** | 5.22 | 1.57 − 17.36 |
|  | Others | 0.162 | 1.76 | 0.80 − 3.89 |

^*^Hazard Ratio, ^**^Confidence Interval for hazard ratio

Bold lettering denotes p value ≤ 0.05

ER (Estrogen Receptor) status became significant when PR (Progestrone Receptor) status and PAM50 were removed from the model.

Supplemental Table S6. Summary of 142 matched samples selected for molecular data analysis

| **Feature** | | **Margin Status** | |
| --- | --- | --- | --- |
|  |  | **Negative (n= 71)** | **Positive (n= 71)** |
| Age Group | Old (60+ years) | 35 | 35 |
|  | Middle Age (40-59) | 34 | 32 |
|  | Young (<40) | 2 | 4 |
|  |  |  |  |
| Menopausal Status | Postmenopausal | 48 | 48 |
|  | Perimenopausal | 2 | 2 |
|  | Premenopausal | 19 | 16 |
|  | Indeterminate | 2 | 3 |
|  | Not Available | 0 | 2 |
|  |  |  |  |
| Race | White | 53 | 45 |
|  | African American | 15 | 15 |
|  | Asian | 1 | 1 |
|  | Not Available | 2 | 10 |
|  |  |  |  |
| Stage | Stage I | 13 | 8 |
|  | Stage II | 28 | 29 |
|  | Stage III | 28 | 26 |
|  | Stage IV | 2 | 8 |
|  |  |  |  |
| PAM50 | LumA | 41 | 40 |
|  | LumB | 11 | 11 |
|  | Basal | 7 | 7 |
|  | Her2 | 9 | 9 |
|  | Normal | 3 | 4 |


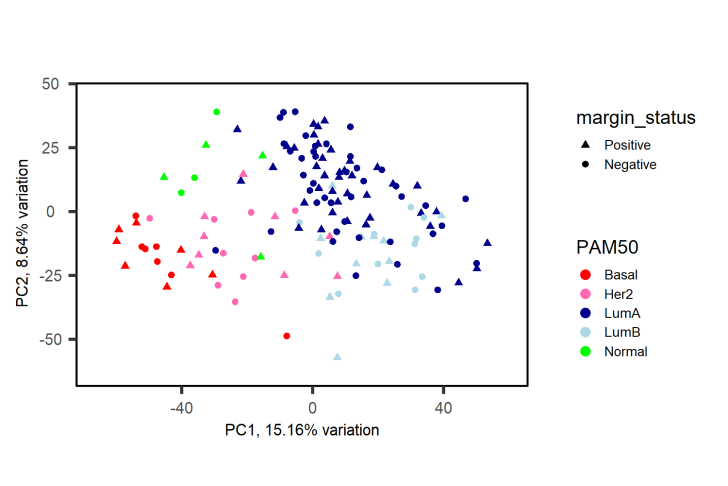


Supplementary **Fig.S2** Principal Component Analysis (PCA) plot for 142 matched samples

Supplementary Table S7. Summary of 29 differentially expressed genes selected through LASSO regression.

| **Gene** | **Description** | **Gene Type** | **FDR^*^** | **FC^**^** |
| --- | --- | --- | --- | --- |
| ARC | Activity Regulated Cytoskeleton Associated Protein | Protein Coding | < 0.001 | 3.87 |
| AMBP | Alpha-1-Microglobulin/Bikunin Precursor | Protein Coding | 0.008 | 2.23 |
| C1orf167 | Chromosome 1 Open Reading Frame 167 | Protein Coding | 0.002 | 3.37 |
| CNGA3 | Cyclic Nucleotide Gated Channel Subunit Alpha 3 | Protein Coding | < 0.001 | 4.74 |
| EEF1A2 | Elongation factor 1-alpha 2 | Protein Coding | 0.033 | 2.32 |
| EPHA6 | Ephrin Type-A Receptor 6 | Protein Coding | 0.007 | 2.80 |
| FOXN4 | Forkhead Box N4 | Protein Coding | 0.024 | 3.17 |
| DIO1 | Iodothyronine Deiodinase 1 | Protein Coding | 0.037 | 2.86 |
| KRT75 | Keratin 75 | Protein Coding | 0.021 | 4.09 |
| SPRR1B | Small Proline Rich Protein 1B | Protein Coding | 0.011 | 5.64 |
| SOX15 | SRY-Box Transcription Factor 15 | Protein Coding | 0.013 | 2.04 |
| STUM | stum, mechanosensory transduction mediator homolog | Protein Coding | 0.003 | 2.80 |
| AC084880.1 | PDGFA associated protein 1 (PDAP1) pseudogene | Pseudogene | 0.017 | 2.07 |
| AC008663.2 | long intergenic non-protein coding RNA 2995 | LncRNA | < 0.001 | 2.98 |
| AC099329.2 | Novel transcript | LncRNA | 0.033 | 2.57 |
| AC004947.1 | Novel transcript | LncRNA | 0.034 | 2.01 |
| ANO3 | Anoctamin 3 | Protein Coding | 0.012 | 0.32 |
| KCNJ6 | Potassium Inwardly Rectifying Channel Subfamily J Member 6 | Protein Coding | < 0.001 | 0.26 |
| PTGDR | Prostaglandin D2 Receptor | Protein Coding | < 0.001 | 0.39 |
| PCP4L1 | Purkinje Cell Protein 4 Like 1 | Protein Coding | 0.003 | 0.40 |
| SPINK1 | Serine Peptidase Inhibitor Kazal Type 1 | Protein Coding | < 0.001 | 0.22 |
| BEND3P1 | BEN Domain Containing 3 Pseudogene 1 | Pseudogene | 0.043 | 0.48 |
| CPHL1P | Ceruloplasmin And Hephaestin Like 1, Pseudogene | Pseudogene | 0.015 | 0.39 |
| AP002001.2 | G1 To S Phase Transition 1 (GSPT1) Pseudogene | Pseudogene | 0.031 | 0.08 |
| LINC01344 | Long intergenic non-protein coding RNA 1344 | LncRNA | 0.037 | 0.44 |
| LINC00589 | Long intergenic non-protein coding RNA 589 | LncRNA | 0.002 | 0.28 |
| AC114296.1 | Novel transcript | LncRNA | 0.022 | 0.40 |
| AF015262.1 | Novel transcript | LncRNA | 0.024 | 0.35 |
| SLC26A4-AS1 | SLC26A4 antisense RNA 1 | LncRNA | 0.004 | 0.38 |

*FDR, False Discovery Rate ** FC, Fold Change

The genes below dotted line are downregulated.
